# Right renal autotransplantation as a kidney-sparing strategy in complex symptomatic angiomyolipoma

**DOI:** 10.1093/jscr/rjaf1110

**Published:** 2026-01-20

**Authors:** Christian Gordon, Francisco Javier Cornejo, Frans Ivan Serpa, Ligia M Redroban, Camila Domenica Valenzuela Molineros, Santiago Muñoz-Palomeque

**Affiliations:** Department of Surgery, Hospital Metropolitano, Quito, 170508, Ecuador, Faculty of Medical, Health and Life Sciences, Universidad Internacional del Ecuador, Quito, 170411, Ecuador; Department of Surgery, Division of Urology, Hospital Metropolitano, Quito, 170508, Ecuador; Department of Surgery, Division of General Surgery, Hospital Metropolitano, Quito, 170508, Ecuador; Department of Internal Medicine, Division of Pathology, Hospital Metropolitano, Quito, 170508, Ecuador; General Physician, University of the Americas, Quito, 170124, Ecuador; Department of Surgery, Hospital Metropolitano, Quito, 170508, Ecuador, Faculty of Medical, Health and Life Sciences, Universidad Internacional del Ecuador, Quito, 170411, Ecuador

**Keywords:** renal angiomyolipoma, renal autotransplantation, bench surgery, nephron-sparing surgery, kidney preservation, ex vivo tumor resection

## Abstract

We present the case of a 34-year-old male with right flank pain. Computed tomography revealed at least six cortical, expansible, fat-density lesions in the right kidney and two similar lesions in the left kidney, consistent with angiomyolipomas. Surgical management consisted of radical right nephrectomy with immediate renal autotransplantation. *Ex vivo* tumour excision was performed under ultrasound guidance and hydrodissection, followed by cortical reconstruction and reimplantation into the ipsilateral iliac fossa. Vascular anastomoses were fashioned end-to-side to the external iliac vessels, and ureteral reimplantation was achieved using the Taguchi technique with double-J stent placement. The postoperative course was uneventful, with preserved renal function. This case highlights the value of multidisciplinary assessment, individualized planning, and meticulous surgical technique to avoid unnecessary radical nephrectomy, particularly in patients with bilateral renal tumors.

## Introduction

Renal angiomyolipoma (AML) is a benign mesenchymal tumor composed of mature adipose tissue, dysmorphic thick-walled blood vessels, and smooth muscle cells. Clinical presentation varies widely, from incidental findings to severe flank pain or life-threatening haemorrhage [1].

Initial management often involves active surveillance; however, treatment depends on tumor size, symptoms, and hemorrhagic risk. Surgical options include partial or total nephrectomy, and in select cases, *ex vivo* tumour resection with renal autotransplantation to preserve nephron mass [1].

Renal autotransplantation was first described by Hardy in 1963 for complex ureteral trauma and later developed by Ota (1967) and Gelin (1971). Although rarely performed, it is an established, technically feasible option for select renal pathologies, including vascular lesions, loin pain–haematuria syndrome, and tumors not amenable to in situ resection [2, 3].

During bench surgery, the graft must be maintained at a stable hypothermic state to minimize ischemia–reperfusion injury and ensure long-term renal function [4]. This case report presents a young patient with bilateral AML managed with right renal autotransplantation as a nephron-sparing strategy, emphasizing the surgical rationale, indications, and technical considerations.

## Case report

A 34-year-old male with no significant past medical history presented with progressive right flank pain. Physical examination and laboratory findings were unremarkable.

**Figure 1:**
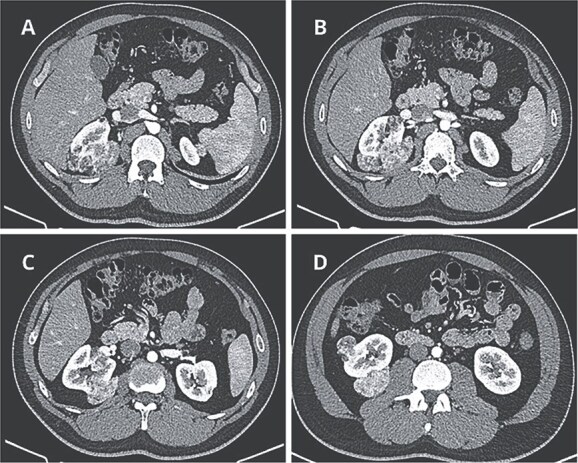
Preoperative CT scan. (A) Axial CT scan showing AML surrounding the right diaphragmatic crus. (B–D) Lesions composed of fat and soft-tissue densities in the upper, middle, and lower poles of the right kidney extending into adjacent retroperitoneal structures.

Contrast-enhanced computed tomography revealed at least six fat-density lesions in the right kidney, predominantly posterior, the largest measuring 3.5 × 4.7 × 6.1 cm, and two smaller lesions in the left kidney—findings consistent with AML (Fig. 1).

Given the tumour location, bilaterality, and limited feasibility of *in situ* resection, a right radical nephrectomy with *ex vivo* tumour resection and renal autotransplantation was planned.

Through a midline incision and Cattell–Braasch maneuver, the right kidney was mobilized. The renal artery, vein, and ureter were meticulously dissected and divided, and the kidney was placed in an ice slush basin.

On the back table, the renal artery was cannulated and perfused with histidine–tryptophan–ketoglutarate (Custodiol®) solution. Under ultrasound guidance, tumor enucleation and aspiration were performed using hydrodissection. Cortical reconstruction was achieved with interrupted 3–0 barbed polydioxanone sutures (Stratafix™), reinforced with Hem-o-lok® clips and oxidized regenerated cellulose for hemostasis.

**Figure 2:**
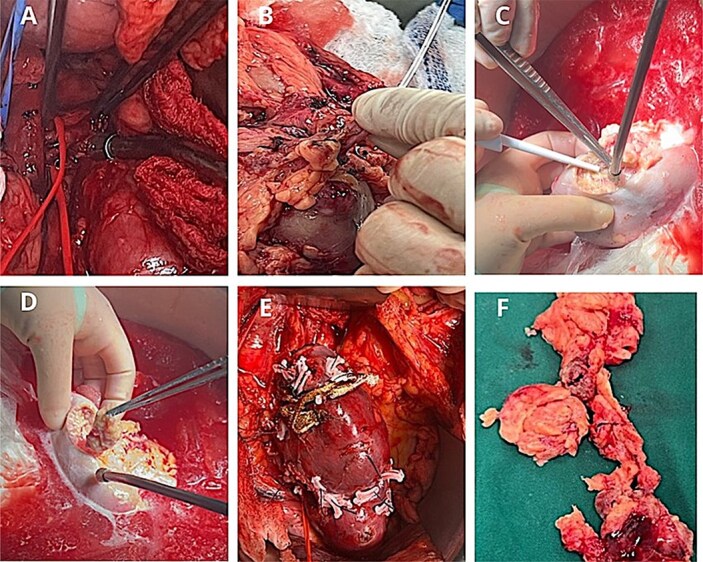
Surgical procedure. (A) Identification of the renal artery posterior to the inferior vena cava. (B) Arterial perfusion with histidine–tryptophan–ketoglutarate (Custodiol®) solution. (C-D) Hydrodissection of the AML under ultrasound guidance. (E) Renorrhaphy with barbed 3–0 Stratafix™ sutures, reinforced with Hem-o-lok® clips and oxidized cellulose. (F) Excised tumour specimens following bench surgery.

The kidney was autotransplanted into the right iliac fossa. Vascular anastomoses were performed end-to-side to the external iliac vessels, and ureteral reimplantation was achieved using the Taguchi technique with double-J stent placement (Fig. 2).

The postoperative course was uneventful. Doppler ultrasound on postoperative day 1 demonstrated preserved perfusion with a resistance index below 0.45.

**Figure 3:**
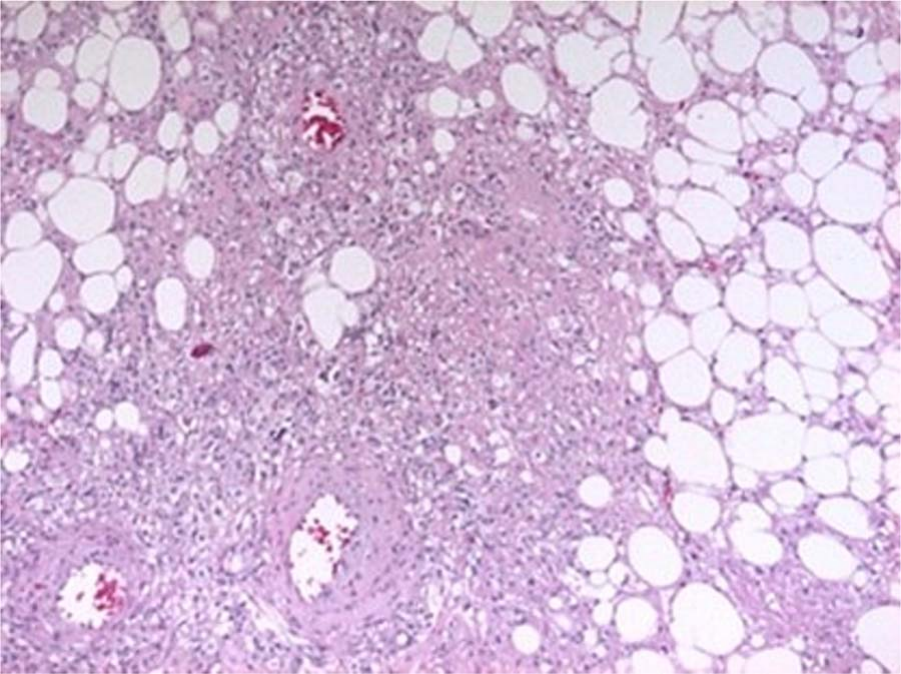
Histopathological features. Spindle-shaped smooth muscle cells without atypia or mitoses, interspersed with mature adipose tissue and variably sized vessels showing transmural hyalinization.

Histopathological examination confirmed AML, showing spindle-shaped smooth muscle cells interspersed with mature adipose tissue and variable-caliber vessels (Fig. 3).

**Figure 4:**
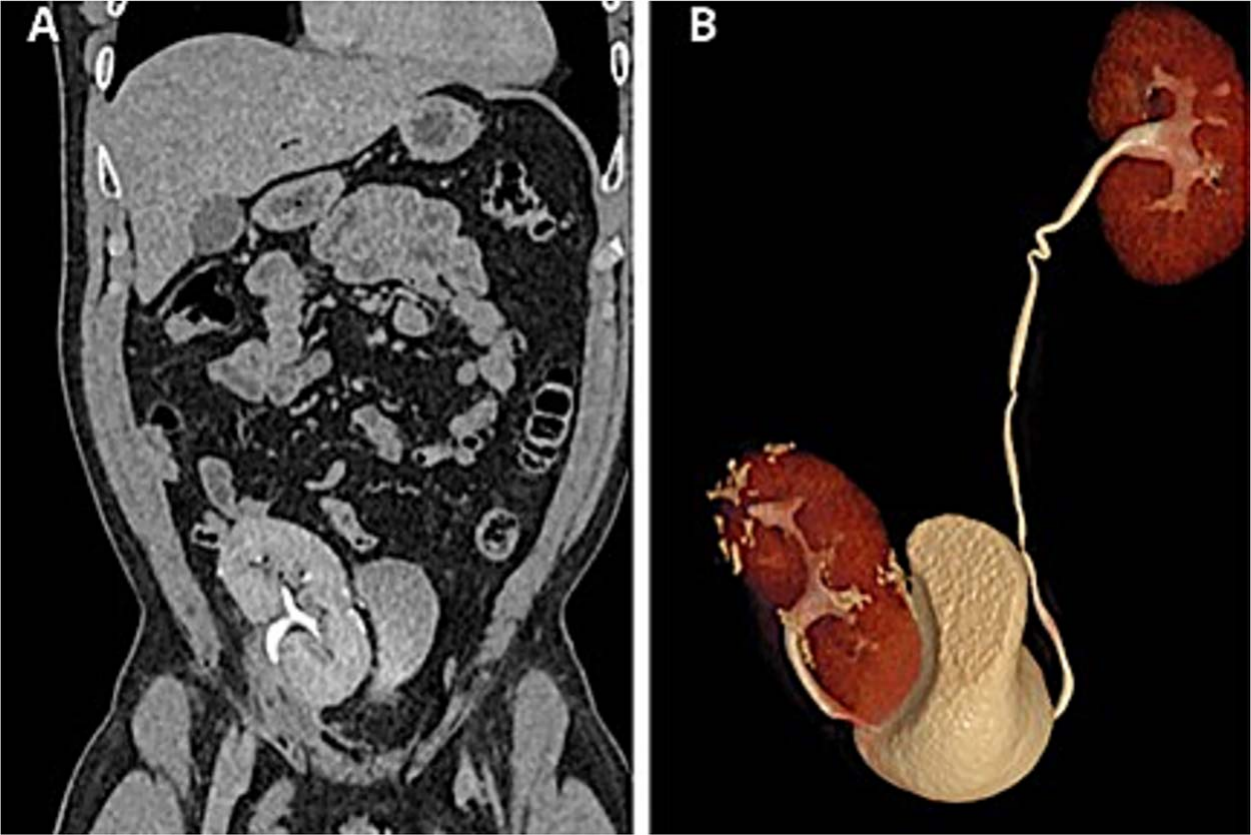
Postoperative CT scan. (A) Autotransplanted right kidney in the iliac fossa demonstrating symmetric contrast excretion and no residual lesions. (B) Normal calyceal morphology and nondilated pelvis; ureters with normal course and caliber.

Renal function remained preserved at 1- and 6-month follow-up, with no evidence of recurrence (Fig. 4).

## Discussion

Renal AML is the most common benign renal tumour. Although typically sporadic, it may also occur in association with tuberous sclerosis complex or pulmonary lymphangioleiomyomatosis, where it exhibits more aggressive growth [1].

Conventional management includes surveillance or selective arterial embolization. However, spontaneous retroperitoneal hemorrhage (Wunderlich syndrome) may necessitate urgent surgery. Nephron-sparing surgery, introduced by Diamond in 1977, enables tumor excision while preserving renal parenchyma, as AMLs rarely invade the renal sinus [4]. Yet, posteriorly located or multiple tumors may limit in situ resection, making *ex vivo* resection with autotransplantation a valuable alternative [2, 5].

Renal autotransplantation allows complete tumour excision while minimizing ischemic injury and preserving renal function. Since its initial use for ureteral reconstruction, indications have broadened to include renovascular disease, ureteral strictures, trauma, and select benign or malignant renal tumors in solitary or bilateral kidneys [2, 3, 6].

In this case, right renal autotransplantation achieved complete resection with preserved renal function and minimal morbidity. Cold ischemia time (90 minutes) remained within acceptable limits for graft preservation. Postoperative Doppler and computed tomography (CT) confirmed satisfactory perfusion and no recurrence at 6 months.

Similar successful outcomes have been reported in the literature, notably by Zheng *et al.* [2], and Woods *et al.* [3], demonstrating that renal autotransplantation is both feasible and durable when performed in experienced centers.

Although technically demanding, this approach should be limited to centers with expertise in microsurgery and kidney transplantation. Prospective studies are warranted to better define its long-term outcomes and optimal patient selection [7].

## Conclusions

Ex vivo tumor resection followed by renal autotransplantation for complex renal AML represents a viable nephron-sparing alternative when in situ resection is not feasible. Successful outcomes depend on careful patient selection, multidisciplinary planning, and surgical expertise to mitigate risks such as vascular thrombosis, urinary fistula, prolonged ischemia, and graft loss.
